# Reflections on the IJHPR’s article collection on dementia

**DOI:** 10.1186/s13584-020-00411-3

**Published:** 2020-10-06

**Authors:** Jill Harrison, Vincent Mor, Susan Mitchell, Ellen P. McCarthy

**Affiliations:** 1 Department of Health Services, Policy, and Practice, School of Public Health, Brown University, Box G-S121 (6), Providence, RI 02912 USA; 2 Providence Veterans Administration Medical Center, Center of Innovation in Health Services Research and Development Service, Providence, RI USA; 3 Hebrew SeniorLife Hinda and Arthur Marcus Institute for Aging Research, Boston, MA USA; 4 Beth Israel Deaconess Medical Center, Department of Medicine, Boston, MA USA

**Keywords:** Dementia care, Alzheimer’s disease, Pragmatic clinical trials, Non-pharmacological interventions

## Abstract

Alzheimer’s disease and Alzheimer’s disease-related dementias (AD/ADRD) constitute a worldwide public health crisis. In light of the AD/ADRD epidemic now existing within the global COVID-19 pandemic, the need for global action to improve dementia care is greater than ever. The article collection “Dementia- an Interdisciplinary Approach,” in the Israeli Journal of Health Policy and Research (IJHPR) highlights the need for interprofessional approaches to improving outcomes for people living with dementia and their care partners, as well as the complexities of conducting dementia care research.

## Dementia- a global epidemic

Alzheimer’s disease and Alzheimer’s disease-related dementias (AD/ADRD) are a group of irreversible and progressive brain disorders that manifest across a continuum of cognitive decline, including difficulty in eating, speaking, problem-solving, making decisions, and memory loss that interfere with a person’s daily activities [1, 2]. Current estimates indicate that 50 million people world-wide live with AD/ADRD [3]. The World Health Organization estimates that the number will triple to more than 150 million people affected globally by 2050 [4]. The global prevalence of dementia varies due to a variety of factors and can be difficult to estimate in some regions [5]. Worldwide, AD/ADRD leaves relatively few populations untouched. It is estimated that someone in the world develops dementia every 3 s [6]. As a disease that exists across a variety of diverse cultures in the world, there is a global population health imperative to develop culturally specific and congruent approaches to provide dementia care.

## Global action to improve dementia care

The article collection “Dementia- an Interdisciplinary Approach,” in the Israeli Journal of Health Policy and Research (IJHPR) [7] highlights the need for multi-dimensional, interprofessional, and internationally collaborative approaches to improve dementia care. The illuminating seven-article series highlights important issues in dementia care including the use of home hospice in cases of advanced dementia [8], ethical, regulatory and legal challenges to conducting clinical research in dementia [9, 10], and the ways in which depictions of dementia in contemporary movies influences the social valuation of the disease [11]. Taken together, these articles emphasize the need for national and global action to improve dementia care.

## What is the NIA IMPACT Collaboratory?

The National Institute on Aging (NIA) IMbedded Pragmatic Alzheimer’s Disease (AD) and AD-Related Dementias (AD/ADRD) Clinical Trials (IMPACT) Collaboratory is part of a national response intended to transform the delivery of dementia care in health care systems in the United States [3]. The IMPACT Collaboratory has created a nationwide infrastructure funded through a cooperative agreement from the National Institute on Aging in order to promote the conduct of embedded Pragmatic Clinical Trials (ePCTs) that evaluate non-pharmacological approaches to care for people living with dementia (PLWD) and their care partners within healthcare systems. This is being accomplished by a coordinated effort of multidisciplinary experts from across the U.S., organized into 10 Working Cores Groups and Teams whose expert members partner with health care system leaders and key stakeholders to develop and disseminate best practice research methods, build investigator capacity through training and knowledge generation, catalyze collaboration among stakeholders, and ensure that dementia research includes culturally-tailored interventions for people from diverse and under-represented backgrounds.

As part of the Collaboratory’s mission to support the design and conduct of ePCTs, a national pilot study award program was established to fund approximately 40 studies over a five-year period with the aim of accelerating tests of nonpharmacologic interventions embedded in everyday clinical practice to improve dementia care [12]. Unlike traditional clinical trials that test interventions under highly controlled conditions, pilot ePCTs test whether dementia care interventions work when they are embedded into everyday healthcare settings under real-world conditions [13]. Pilot ePCTs have tremendous potential to close the science-to-service gap, as described by Golander in the description of mini-research projects as a mechanism to improve the quality of dementia care [14] and by Sternberg and colleagues in their pilot study of home hospice for severe dementia [8].

ePCTs are designed to inform clinical and policy-level decisions about dementia care that works in the real-world. Co-authors Bentur & Sternberg [15] explore several ways in which ePCTs bridge research and practice in their discussion of top-down and bottom-up approaches to optimize the relevancy of trials to stakeholders’ everyday lives. The convergence of policy/regulatory/researcher-initiated research (referred to as top-down approaches), with interventions initiated by clinical and lived experience and knowledge of being immersed in daily dementia care (referred to as bottom-up approaches) is the dynamic needed not only to improve the person-centeredness of dementia research [16], but also its uptake, adoption, and implementation by the intended end-users [17].

## An epidemic within a pandemic

The IJHPR article collection on dementia was curated prior to the insidious global spread of the novel Coronavirus. Since early 2020, the global dementia epidemic now exists within the COVID-19 pandemic. AD/ADRD and COVID-19 are both international public-health crises that disproportionately impact older adults and, for both, there are no known effective medical treatments. Non-drug interventions, such as masks, social distancing, and hand hygiene are being deployed world-wide in the fight against COVID-19. Some of these critical infection control practices have unintended consequences for dementia care. For example, people living with dementia may be confused at the sight of care partners in masks and may refuse or be unable to wear one themselves. Social distancing and quarantine orders to prevent virus spread compound social isolation of people living with dementia, a factor that is associated with incidence and severity of AD/ADRD [18].

We are learning more about the experiences of PLWD in long-term care facilities during COVID-19, in which many residents have been restricted from seeing family and friends. However, we know very little about the experiences of PLWD living alone in the context of COVID and about the pandemic’s impact on caregivers living in the community. These include the impact on PLWD in alternative care arrangements, such as the shared housing arrangements in Germany, described by Gräske, Schmidt, and Wolf-Ostermann in their article in the IJHPR collection [19]. The caregiver burden for Alzheimer’s, in Israel and other nations, was already extremely high before the pandemic [15]. The financial burdens and stress for caregivers, often referred to as “second patients,” are likely to compound as a result of a variety of COVID-related factors such as social isolation, economic hardship associated with changes to employment, limited access to supportive services and the intersection of multiple caregiving roles as a result of school closures. More research is needed to build knowledge about living with dementia and caregiving in the context of COVID, including its impact on health equity [20] and how societies can build their capacity to anticipate and address disparities.

## Conclusion

The issues addressed in the IJHPR article collection have never been more salient and will undoubtedly continue to have international resonance as the world looks to respond to the unmet needs of people living with dementia and care partners. The application of the issues raised in the article collection to other settings and other cultures will be a critical agenda for research and practice for years to come. In light of the AD/ADRD epidemic now existing within a global pandemic, the need for global action to improve dementia care has never been greater. The current intersection of these health crises is a much-needed wake-up call as we prepare for the certainty of an uncertain future. Will we be better prepared the next time?

## Data Availability

N/A.

## Abbreviations

PLWD: Person living with dementia
ePCT: Embedded pragmatic clinical trial
